# Effect of Nonviral Plasmid Delivered Basic Fibroblast Growth Factor and Low Intensity Pulsed Ultrasound on Mandibular Condylar Growth: A Preliminary Study

**DOI:** 10.1155/2014/426710

**Published:** 2014-05-20

**Authors:** Harmanpreet Kaur, Hasan Uludağ, Tarek El-Bialy

**Affiliations:** ^1^Department of Dentistry, Faculty of Medicine and Dentistry, Medical Science Graduate Program, University of Alberta, Edmonton, AB, Canada T6G 2E1; ^2^Department of Chemical and Materials Engineering, Faculty of Engineering, University of Alberta, Edmonton, AB, Canada T6G 2E1; ^3^Department of Dentistry, Faculty of Medicine and Dentistry, University of Alberta, Edmonton, AB, Canada T6G 2E1

## Abstract

*Objective*. Basic fibroblast growth factor (bFGF) is an important regulator of tissue growth. Previous studies have shown that low intensity pulsed ultrasound (LIPUS) stimulates bone growth. The objective of this study was to evaluate the possible synergetic effect of LIPUS and local injection of nonviral bFGF plasmid DNA (pDNA) on mandibular growth in rats. *Design*. Groups were control, blank pDNA, bFGF pDNA, LIPUS, and bFGF pDNA + LIPUS. Treatments were performed for 28 days. Significant increase was observed in mandibular height and condylar length in LIPUS groups. MicroCT analysis showed significant increase in bone volume fraction in bFGF pDNA + LIPUS group. Histomorphometric analysis showed increased cell count and condylar proliferative and hypertrophic layers widths in bFGF pDNA group. *Results*. Current study showed increased mandibular condylar growth in either bFGF pDNA or LIPUS groups compared to the combined group that showed only increased bone volume fraction. *Conclusion*. It appears that there is an additive effect of bFGF + LIPUS on the mandibular growth.

## 1. Introduction

Bone remodelling is a continuous process of bone formation and resorption to maintain bone shape and function. But many conditions like tumors, trauma, skeletal abnormalities, and congenital disorders can compromise this dynamic process [1]. 700 out of 6000 known congenital syndromes involve craniofacial defects which include but not limited to Treacher Collin Syndrome, and Pierre Robin Syndrome [2]. These problems not only affect the social life but also have the psychological effects on the affected individuals [3–5]. The available treatments of underdeveloped lower jaw in these cases usually include orthopedic surgery, bone grafting, and distraction osteogenesis in addition to orthodontic treatment and speech and behavioral management [6]. All these treatment modalities have various limitations such as lack of required bone volume, donor site morbidity, long procedure time, graft resorption, disease transmission, and known surgical complications. Due to all these limitations, a nonconventional form of treatment like gene therapy may be a hope to enhance or stimulate lower jaw growth nonsurgically.

The process of bone formation takes place by two methods: intramembranous and endochondral bone formation. In endochondral ossification, the chondrocytes present in the cartilage undergo morphogenesis and calcification by the invasion of blood vessels which results in the new bone formation [7]. Hence the vascularization is an essential step in the endochondral bone formation. Growth factors like vascular endothelial growth factor (VEGF) and bFGF play an important role in the process of new blood vessel formation. Many growth factors have been studied for their regulatory effect in the cell activities like adhesion, proliferation, and differentiation in epithelium, bone, connective tissue, and the nerves [8]. Basic fibroblast growth factor (bFGF) belongs to a family of 22 members. It is present in bone matrix and it plays an important role in the initial vascularization for the endochondral bone formation [9, 10]. bFGF is a potent cytokine that not only helps in angiogenesis but also has a stimulatory role in osteogenic differentiation of preosteoblasts [11], in limb development, and in bone fracture healing [10]. It exhibits its biological function by binding and activation of FGF receptors 1, 2, and 3. bFGF has important effects on bone formation of the facial region. One study [12] showed that blocking of bFGF leads to the prevention of bone formation at the craniofacial suture sites and study by Hamada et al. [13] showed that receptors of FGF family are present in the condylar cartilage which helps in the differential growth of the condylar cartilage. Rabie et al. [6] have successfully studied the effect of adenovirus delivered VEGF on the mandibular condylar growth.

Gene therapy is a fast developing technology that is defined as the treatment of disease by the transfer of the genetic material into the cells in the form of small DNA or RNA fragments and has been used for the treatment of diseases like genetic disorders [14], cancer [15] and neuro-degenerative disease [16]. The expected success of gene therapy depends on its delivery system. For the delivery of the gene, either virus or nonvirus vectors may be used as carriers. Viral vectors provide efficient gene delivery to the targeted tissue cells and longer gene expression. But due to safety concerns associated with viral vectors like immunogenicity and oncogenicity [17] and death of a patient in 1999 after adenoviral mediated gene therapy due to disseminated intravascular coagulation and multiple organ failure [18], a nonviral vector is a preferred gene delivery approach. Nonviral gene delivery involves the transfer of the genetic material either by direct injection of the plasmid or by physical or chemical methods. Direct delivery of the plasmid DNA (pDNA) is the most simple and the most convenient method of the gene delivery. Electroporation and sonoporation are two examples of the physical methods used in the gene delivery.

Ultrasound is an acoustic pressure or energy that propagates through the media in the form of waves having the frequency above the human hearing range. The low intensity ultrasound is studied for its role in drug delivery into solid tumor [19], gene delivery to the target tissue [20–22], treatment of bone fracture, distraction osteogenesis [23–26], reduction of root resorption after tooth movement [27], and also the growth of the mandibular condyle [28–30]. Ultrasound application for the treatment of bone fracture has been approved by Food and Drug Association, USA. The exact mechanism is still unclear; however, it has been suggested that the effects of LIPUS may be physical or piezoelectric in nature [31]. Recently, LIPUS has been used as one of the physical methods for the gene delivery by using intensities ranging from 0.4 W/cm^2^ [32] to 1 W/cm^2^ [21] to 2 W/cm^2^ [22]. In a study by Zhou et al. [33] for the gene transfection, in the* in vitro* procedure intensity applied was 0.75 W/cm^2^ and for* in vivo* the intensity used was 2 W/cm^2^. However, these intensities might lead to tissue heating which usually is undesirable. So, lower intensities of the ultrasound might be more desirable and also effective in delivering nonviral victors-loaded bFGF to stimulate mandibular condylar growth.

The hypothesis of this pilot study was that bFGF combined with LIPUS would enhance the mandibular growth. The objectives of this study were to explore the possible effect of the local injection of bFGF plasmid and daily low intensity pulsed ultrasound (LIPUS) application on the mandibular condylar growth.

## 2. Materials and Methods

### 2.1. Animal Care and the Experimental Design

A total of fifteen late adolescent (~200 gm) adult Sprague Dawley rats were obtained from the Biosciences Laboratory, University of Alberta, Edmonton. All the animal procedures were performed according to the guidelines of the Canadian Council on Animal Care and the study was approved by Animal Welfare Committee at the University of Alberta. Before the procedure, the rats were allowed to acclimatize for a period of 7 days. The rats were housed in pairs in clean cages and were allowed free access to the standard commercial rat chow (Lab Diet, St. Louis, MO, USA) and tap water. The rats were randomly divided into 5 groups (*n* = 3). Group 1 was the control, Group 2 was injected with blank plasmid (25 *μ*gm gWiz) on the first day of the experiment, Group 3 was injected with 25 *μ*gm bFGF pDNA (description of the plasmid is provided below) on the first day of the experiment, Group 4 received 20 min of LIPUS for the next 28 days, and Group 5 was injected with 25 *μ*gm bFGF pDNA on the first day of the experiment and received LIPUS application for 20 min for 28 days. In all groups, left mandibular condyle was used as the experimental side, while the right side was left as internal control. The treatment side of each animal was shaved and coupling gel was applied to ensure the wave propagation. The prepared solutions corresponding to each group were injected to the posterior attachment of the mandibular condyles in the experimental side using (1/2) cc U-100 insulin syringe with attached 28(1/2) gauge needle (Becton-Dickinson & Company, Franklin Lakes, NJ, USA) according to the previously reported technique [6, 34]. Before injection, aspiration was performed to make sure that the needle is not into a blood vessel. The content was released slowly over a period of one min to prevent any damage to the surrounding structures. During the ultrasound application, the animals were under inhalation anesthesia of 2.5% isoflurane with 100% oxygen. Twenty-four hours after the final application of LIPUS, the animals were euthanized by using asphyxiation in CO_2_ chamber. The mandibles were carefully dissected and fixed in 4% formalin (Sigma-Aldrich, Oakville, Ontario, CA) for 24 hours at room temperature.

### 2.2. Plasmid Material

The plasmids used in this study were a commercially available blank plasmid (gWiz) encoding no functional genes and a plasmid (pFGF2-IRES-AcGFP) encoding for both bFGF and green fluorescence protein (GFP) with an internal ribosomal entry site (IRES). The construction and preparation of the latter plasmid were described in Clements et al. [11]. The plasmids were mixed with a lipopolymer (linoleic acid substituted 2 kDa polyethyleneimine [35] at plasmid: polymer ratio of 1 : 5 in 0.15 M NaCl (25 *μ*g plasmid to 125 *μ*g polymer in 100 *μ*L injection volume per rat)). The plasmid/polymer mixtures were allowed to incubate for 30 minutes before injection into rats.

### 2.3. Ultrasound Application and Calibration

The ultrasound device was provided by Smile Sonica Ltd., Edmonton, Alberta, Canada. The transducer has an emitting surface area of 1.5 cm^2^ (12 mm × 13 mm) and generated 200 microsecond burst of 1.5 MHz sine wave repeating at 1 kHz that delivered temporal averaged intensity of 30 mW/cm^2^. The ultrasound device was calibrated at the beginning and at the end of the experiment confirming that the ultrasound device provided constant power output and maintained the desired parameters during the experiment.

### 2.4. Anthropometric Measurements of the Mandible

**Figure 1:**
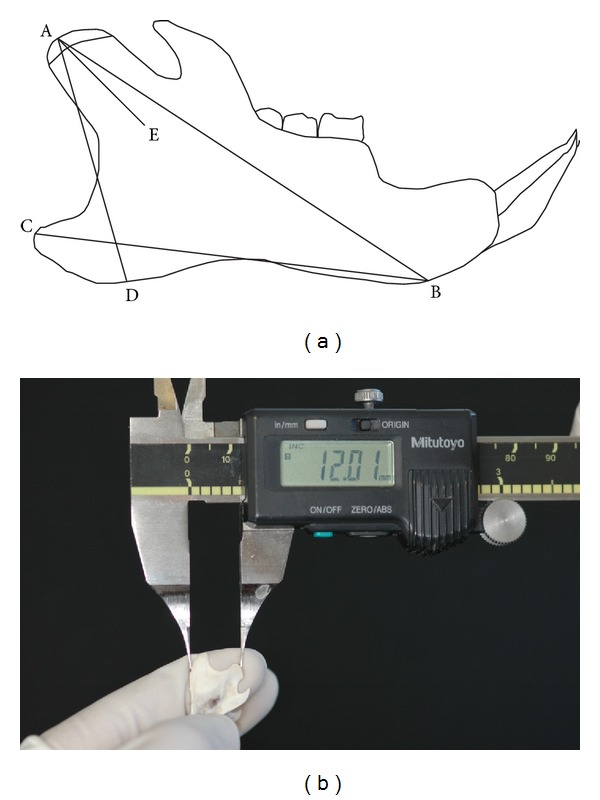
(a) The diagram illustrating the anthropometric points and linear measurements of the mandible (for definition, see Table 1). (b) The anthropometric measurement of the extracted rat mandible with the help of digital vernier caliper.

The extracted mandibles were divided at the symphyseal junction into two hemimandibles.  Figure 1(a) shows the points and the linear measurements of the mandible. The mandibles were measured using a digital caliper (Figure 1(b)). The description of the points and the parameters are presented in Table 1 [36].

### 2.5. Micro-CT Imaging

**Figure 2:**
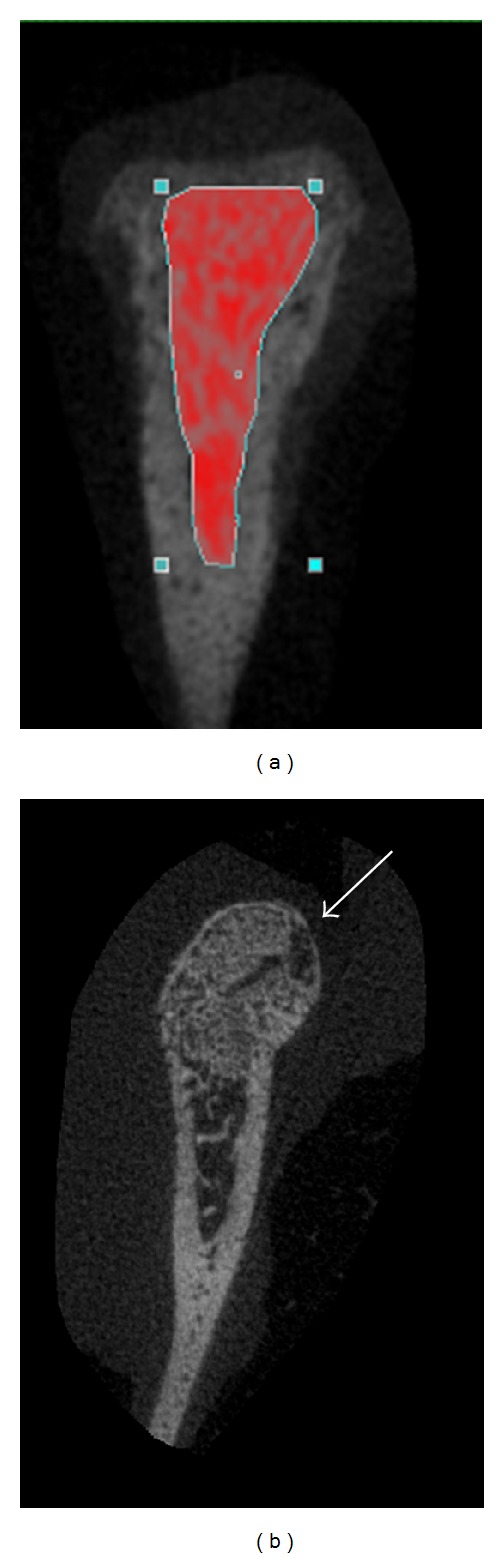
Transaxial view of the Micro-CT scan. (a) The region of interest was manually drawn to separate trabecular bone from the cortical bone of the condyle and was analysed later using Micro-CT Analyser (Skyscan, NV, BE). (b) The new bone formation in the bFGF treated group on the mandibular condylar head.

The hemimandibles were scanned using Micro-CT imager, Skyscan 1076, Skyscan NV, Belgium, with resolution of 18 *μ*m at 0.5° step increments with 1180 msec exposure time. The tube voltage and the current were 70 kV and 139 *μ*A, respectively. The raw image data were reconstructed using modified Feldkamp back projection algorithm with the cross section threshold of 0.00–0.04 using NRecon reconstruction software (version 1.4.4, Skyscan NV, Belgium). The analysis of the microarchitecture was done on the vendor supplied CTAN software (Skyscan NV, Belgium). The region of interest (ROI) was selected on the condylar trabecular bone (Figure 2). Bone mineral density (BMD) was determined based on the linear correlation between CT attenuation coefficient and bone mineral density using calibrated phantom. The parameters evaluated from the scans were bone volume fraction (BV/TV), bone volume (BVol), and bone mineral density (BMD).

### 2.6. Histology and Histomorphometric Analysis

The mandibles were decalcified using Cal-EX II (Fisher Scientific, Ottawa, CA) (formaldehyde 1.03 M/L, formic acid 2.56 M/L) for about 2 weeks. The samples were processed into paraffin blocks and sectioned at a thickness of 6 *μ*m and were stained with hematoxylin and eosin stain. Six samples were obtained from each hemimandible and the images were obtained using Leica fluorescent digital microscope with CCD digital camera (Leica, Wetzlar, Germany) at a 20x magnification. The analysis of the images was performed using RS Image software 1.73 (Photometric, Roper Scientific Inc., Tucson, AZ, USA). The condylar cartilage was divided into 4 zones: resting, proliferative, hypertrophic, and erosive. Proliferative layer is composed of densely packed mesenchymal cells with high nuclei and cytoplasm ratio. Hypertrophic layer is subdivided into mature chondrocytes and hypertrophic chondrocytes. The cells are larger than the cells in proliferative layer. In this study the mature and hypertrophic chondrocytes were analysed together as the hypertrophic layer. The proliferative and hypertrophic layers were studied according to their histological characteristics. Cell number and the width of the proliferative and hypertrophic layers were measured. The readings from the six slides of each sample were then averaged to get the final reading for every sample.

### 2.7. Statistical Analysis

The data were collected and processed using SPSS 19.0. by Kruskal-Wallis nonparametric test for analysis of all the five groups because of the relative small sample size. For comparison between groups, Mann-Whitney* U* test was used. The mean and standard deviation are presented in the bar graph for each variable. Statistical significance level was set at *P* < 0.05.

## 3. Results

No inflammation or irritation was noted at the injection site. No reduction in weight was noticed during the treatment phase.

### 3.1. Anthropometric Analysis

**Figure 3:**
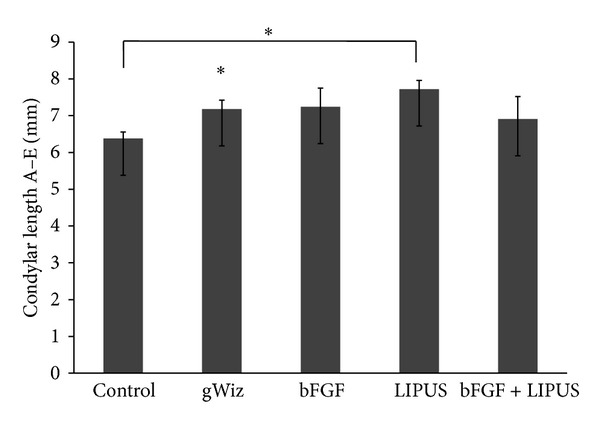
The bar chart of the length of the condylar process (A–E) among the five groups showing increase in the length in the LIPUS treated group {* = *P* ≤ 0.05}.

**Figure 4:**
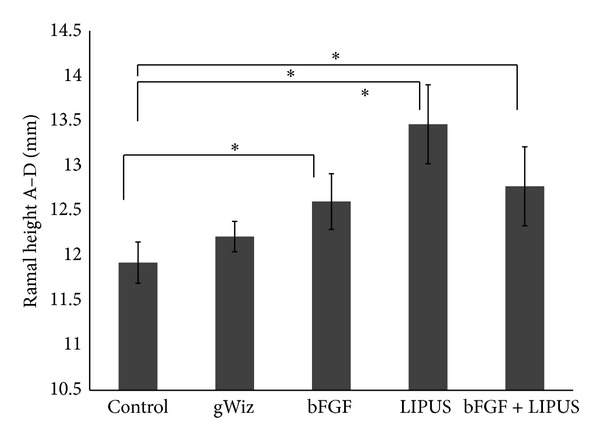
The bar chart of the ramal height (A–D) of the mandible among the five groups showing increase in the height of the mandible in LIPUS treated group {* = *P* ≤ 0.05}.

**Figure 5:**
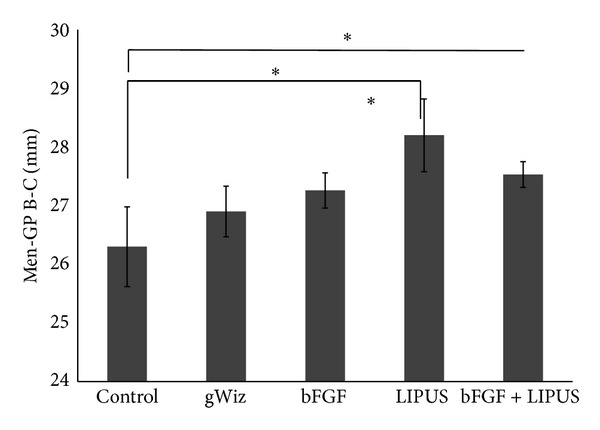
The bar chart of the Men-GP (B-C) of the mandible among the five groups showing increase in the height of the mandible in LIPUS treated group {* = *P* ≤ 0.05}.

Linear measurements of Condyle-GoT (ramal height, A–D), men-GP (mandibular base, B-C), and the condylar point-mandibular foramen (condylar process, A–E) showed statistically significant increase in the LIPUS. In Figure 3, there is significant difference between control and LIPUS treated group and control and bFGF group (*P* < 0.05). Ramal height (A–D) showed statistical increase in all the treatment groups compared to the control group (*P* < 0.05). There is also significant difference between bFGF and LIPUS groups (Figure 4). Men-GP (B-C) showed statistical significant increase in the LIPUS group compared to the control group (*P* < 0.05) and compared to bFGF group (*P* < 0.05). Also, men-GP (B-C) showed statistically significant difference between control and bFGF + LIPUS group (Figure 5) while the linear measurement of the condylar point-men (length of the mandible, A-B) showed no statistically significant difference (data not shown). Overall, by comparing the means of the groups, LIPUS treated group showed the maximum increase in the anthropometric measurement followed by the combination treatment of bFGF + LIPUS followed by bFGF treated group.

### 3.2. Micro-CT Analysis

**Figure 6:**
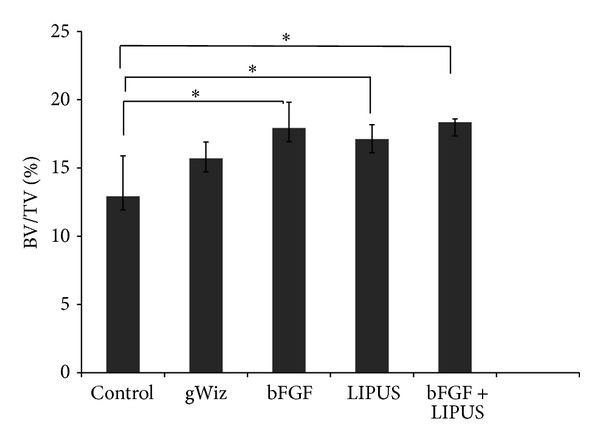
The bar chart of the bone volume fraction of the mandible condyle showing increase in bone volume fraction in the combination group (bFGF + LIPUS) {* = *P* ≤ 0.05}.

Of all the variables measured in the Micro-CT analysis, only bone volume fraction showed significant difference among all the five groups (*P* < 0.05) (Figure 6). On the other hand, bone volume and BMD showed no significant results (*P* > 0.05). By comparing the means of bone volume and BMD of the groups, the combination treatment of bFGF + LIPUS showed the highest mean followed by bFGF group and LIPUS treated group (Table 2).

### 3.3. Histomorphometric Analysis

**Figure 7:**
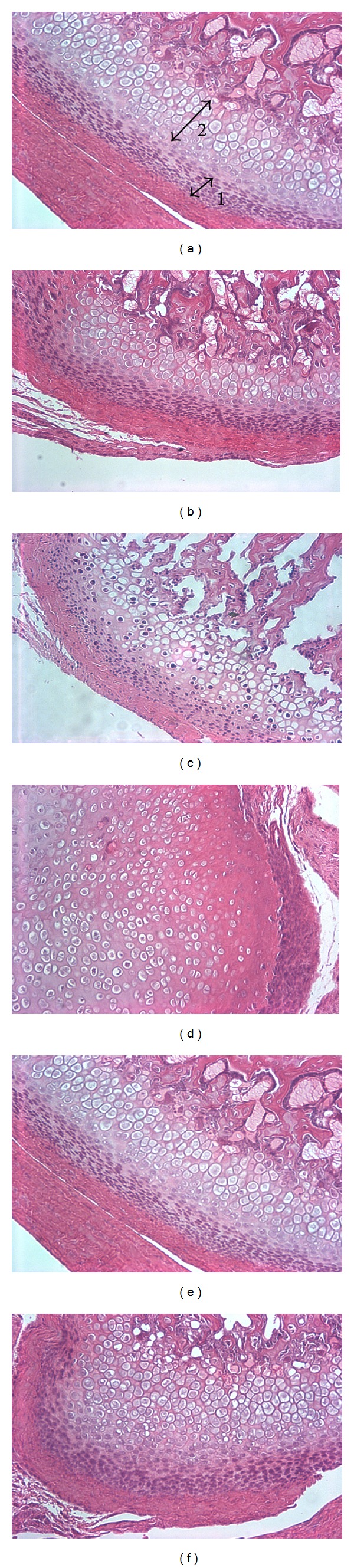
H&E stained sections of the articular surface of the condyle in the treatment groups seen in 20x magnification. (a) The proliferative layer marked by arrow 1 and the hypertrophic layer marked by arrow 2. (b) Control group. (c) gWiz group. (d) bFGF group (e) LIPUS group. (f) bFGF + LIPUS group.

**Figure 8:**
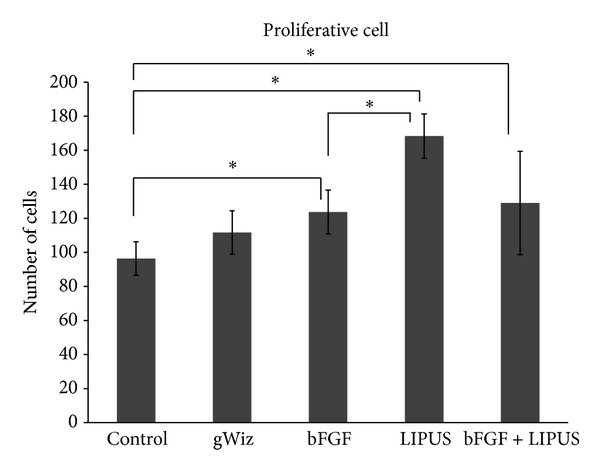
The bar chart shows increase in proliferative cell count for the LIPUS treated group {*P < 0.05}.

**Figure 9:**
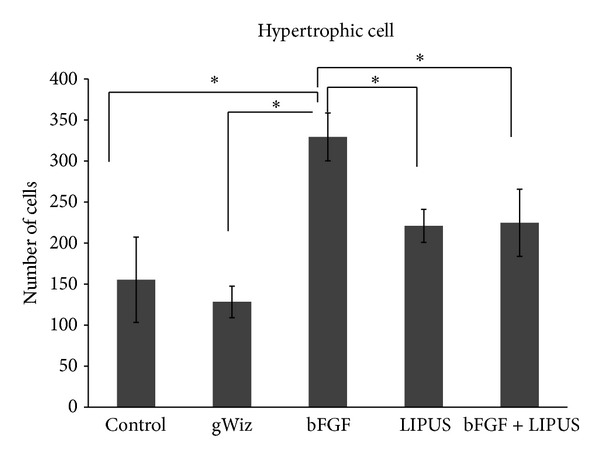
The bar chart depicts the result for the hypertrophic cell count of the condyle showing increase in the cell number in the bFGF treated group {*P < 0.05}.

**Figure 10:**
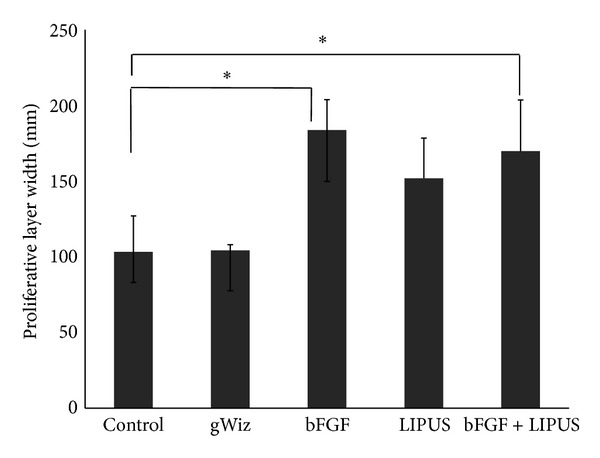
The bar chart depicts the result for the width of the proliferative layer of the condyle showing increase in the width in the bFGF treated group {* = *P* ≤ 0.05}.

**Figure 11:**
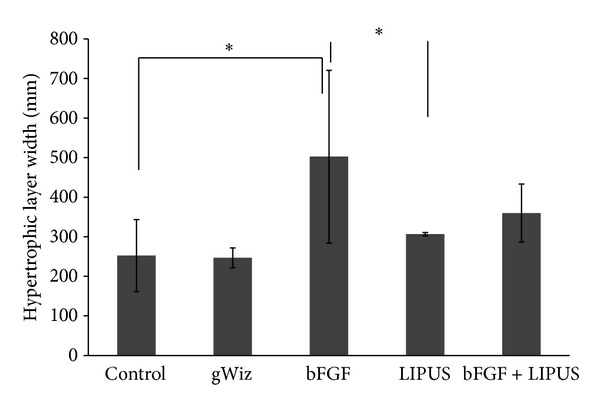
The bar chart depicts the result for the width of the hypertrophic layer of the condyle showing increase in the width in the bFGF treated group {* = *P* ≤ 0.05}.

Cellular morphological evaluation revealed that the LIPUS treated group showed increase in the cell size of the hypertrophic layer while the bFGF treated group showed increase in the number of the cells in hypertrophic layer but the cell size was small as compared to LIPUS treated group. Moreover, the cells in both layers were loosely packed in bFGF group as compared to the LIPUS treated and combined treatment groups (Figure 7). No significant difference was found between LIPUS and bFGF + LIPUS groups in any of the variables. The mean and the standard deviation of the measured variables are shown in Figures 8, 9, 10, and 11. By comparing the means of the groups, LIPUS treated group showed the highest cell number in the proliferative layer while bFGF treated group showed the highest increase in the cell number in hypertrophic layer and increase in the width of the proliferative and hypertrophic layers. There was significant increase in the proliferative layer in bFGF and bFGF + LIPUS compared to the control group (Figure 10).

## 4. Discussion

This study was performed to explore if there is any stimulatory effect of the nonviral plasmid delivered bFGF with or without LIPUS treatment on the condylar cartilage and on mandibular growth. In our study, we investigated the effect of plasmid delivered bFGF alone and in combination with LIPUS on the mandibular condyle growth. In the present study, microbubble was not added to the plasmid solution compared to the previous studies and the ultrasound parameters used in this study were different from the previous studies as pulsed low intensity ultrasound is used while other studies used continuous wave [37–39]. We also injected the plasmid into the posterior attachment of the condyle to study the effect on the bone growth. The study did not intend to use LIPUS to enhance gene transfection; bFGF plasmid was used to possibly enhance the mandibular growth directly and the main objective of this study was to find if there is any synergetic effect of the combining of both techniques or not. The results of this study showed that bFGF and LIPUS alone can have a positive effect on mandibular condylar growth as seen in the histomorphometric and anthropometric measurement, respectively, while the combination therapy of bFGF and LIPUS showed only increase in the bone volume fraction. Histomorphometric analysis showed significant increase in the proliferative cell count and the width of the proliferative and hypertrophic layers in the bFGF treated group. The proliferative layer consists of undifferentiated mesenchymal cells while the hypertrophic layer consists of mature chondrocytes which is important for the condylar growth [40]. The chondrocytes undergo hypertrophic changes in this layer and the first sign of calcification is present in this layer, too. Also the qualitative study of the slides was different in the treated groups. The group treated with LIPUS showed larger cell size compared to the group treated with bFGF which were smaller and loosely packed. The possible reason for the higher values of the histological parameters could be due to the close proximity of the area to the injection site which lead to bone formation on the condylar head (Figure 2(b)). The difference between LIPUS and bFGF histological sections could be due to the fact that LIPUS was applied to the whole condyle, while bFGF was applied to the posterior part only. Also, this difference might explain the slight increase, while insignificant, in condylar length in LIPUS group compared to plasmid group.

Being a pilot study, the objective was only to study the effect of bFGF alone and in the combination with LIPUS and to study the bone growth on the mandibular condylar head as a proof or principle, and hence no test was conducted to check for the presence of the plasmid at the end of the treatment or assess the duration of gene expression during the study period. These questions warrant more extensive studies that are planned in the future in the authors' labs.

Anthropometric measurement demonstrated that the LIPUS treated group showed the best result among all the groups. These results are in agreement with the previous studies where the linear measurements increased after LIPUS application [24, 28]. Although the exact mechanism of action for the LIPUS is still unclear; however, it has been suggested that the effects of LIPUS may be physical or piezoelectric in nature [31]. LIPUS produces vibration forces in all tissue components, both intracellular and extracellular. These vibrations cause movements of the particles in the tissue which causes mechanical stimulation. Therapeutic ultrasound using low intensities (20–50 mW/cm^2^) causes small increase of temperature which may affect the cellular mechanism that may cause bone remodelling and growth. According to Wolff's law, the bone in a healthy person and animal will adapt to the load it is placed under. If the loading on the particular bone increases the bone will remodel itself over the time to be stronger to resist the loading [41]. Bone is piezoelectric in nature and remodels itself according to the functional demands and environmental forces [42]. Ultrasound produces physiological mechanical stress in the bone that causes its deformation. This deformation causes the generation of potential differences in the cells which causes bone remodelling [43]. Low intensity ultrasound produces nonthermal effect which causes stable cavitation, microstreaming, and mechanical effect on the cell membrane [44]. Studies have shown that LIPUS enhance the exchange of ions intracellularly and extracellularly [45], change in the second messenger concentration which lead to alteration in gene expression for the cartilage and bone specific genes [46], increase in intracellular concentration of calcium in chondrocytes [47], and increase in the angiogenesis related cytokines [48]. The lower intensity pulsed ultrasound used in this study is less likely to produce cavitation without introducing microbubble. Future studies may aim at evaluating this effect both* in vitro* and maybe* in vivo*. It is to be noted that the used LIPUS was not intended to perform sonoporation for the gene delivery. This might explain the nonstatistical difference between LIPUS and bFGF + LIPUS groups.

In the Micro CT analysis, our study showed that the combination treatment of bFGF and LIPUS has significant effect on the bone volume fraction while all other variables, that is, the bone volume and BMD, were nonsignificant. Although on comparing the means, still the combination therapy showed better results as compared to the other treatment groups except that BV/TV (bone volume fraction) did not increase much in the combination therapy compared to either treatment separately. This might be explained as that there might be minimum synergetic effect between LIPUS and bFGF at the study end point (28 days) that is reflected on the bone formation which may warrant future long term study. The bone volume fraction is the ratio of bone volume to the total volume of the region of interest and plays an important role as an interpreter of the mechanical properties of the bone. In this study only the trabecular bone of the condylar process was studied by manually drawing the region of interest to separate the trabecular bone from the cortical bone. The reason for selecting the trabecular bone was that it has high turnover rate as compared to the cortical bone and is the major site to detect the early changes after the therapy [49]. An explanation of these results could be that, after 28 days of treatment, bFGF injection leads to bone formation at the site of the injection and the LIPUS application leads to early maturation and these factors are combined in the bFGF + LIPUS group while the other treatment groups could still be in the early phases of the growth. This speculation needs to be further evaluated by future studies. The difference between histological and MicroCT data among the groups could be due to the short period of treatment (28 days) which could have an effect at the cellular level while the gross anatomy effect might need longer treatment/observation time. This hypothesis also suggests future evaluation.

In conclusion, within the limitations of this pilot study, the present preliminary study indicates that the combination treatment of bFGF and LIPUS has selective effect on the mandibular condyle growth. More studies are needed not only to be with larger sample size but also to find the molecular, cellular basis and the long term study with time interval.

## Figures and Tables

**Table 1:** Description of the anthropometric points and linear measurements.

	Description
Points	
Condylar point (A)	The most posterior and superior point on the mandible condyle.
Menton (B)	The most inferior point on the mandibular symphysis.
Gonion point (C)	The most posterior point on the bony contour of the gonial angle of the mandible.
Gonion tangent point (D)	Assuming that the mandible is placed on a plane. The point of the mandibular gonion at its junction with that plane.
Mandibular foramen (E)	The point of entry of mandibular nerve and blood vessels into the mandibular canal.
Linear measurement	
Menton-condylar point (A-B)	Total mandibular length.The distance measured between menton and condylar points.
Menton-gonion point (B-C)	Length of mandibular base.The distance measured between menton and gonion points.
Condylar-GoT (A–D)	Ramus height.The distance measured between condylar and gonial tangent points.
Condylar process length (A–E)	The distance measured between mandibular foramen to condylar points.

**Table 2:** Mean and standard deviation of BMD and bone volume.

	Control	gWiz	bFGF	LIPUS	bFGF + LIPUS
BMD					
Mean	0.0048	0.0053	0.0078	0.0063	0.0086
Std dev.	0.001	0	0.003	0	0.001
BVol					
Mean	0.0926	0.09	0.109	0.1003	0.1069
Std dev.	0.017	0.038	0.006	0.021	0.008
